# Tumour angiogenesis in Epstein-Barr virus-associated post-transplant smooth muscle tumours

**DOI:** 10.1186/2045-3329-4-1

**Published:** 2014-01-07

**Authors:** Danny Jonigk, Nicole Izykowski, Lavinia Maegel, Eileen Schormann, Britta Ludewig, Hans Kreipe, Kais Hussein

**Affiliations:** 1 Institute of Pathology, Hannover Medical School (MHH), Carl-Neuberg-Str. 1, D-30625 Hanover, Germany

**Keywords:** PTSMT, Post-transplant smooth muscle tumours, EBV, Angiogenesis, Tumour

## Abstract

Epstein-Barr virus (EBV)-associated post-transplant smooth muscle tumours (PTSMT), are rare complications following organ/stem cell transplantation. Despite the mainly benign behaviour of PTSMT, alternative therapies are needed for those patients with progressive tumours. In tumours not approachable by surgery or reduction of immunosuppression, the angiogenic microenvironment might be a potential target of therapy, an approach that is well utilised in other soft tissue neoplasms. In a previous study, we evaluated the expression of EBV-related genes and the microRNA profile in PTSMT, but so far the characteristics of angiogenesis in PTSMT are not known. Therefore, the aim of this study was to evaluate the expression pattern of angiogenesis-related genes in PTSMT, in order to identify potential target molecules for anti-angiogenic therapy.

PTSMT (n = 5 tumours) were compared with uterine leiomyomas (n = 7). Analyses included real-time PCR of 45 angiogenesis-associated genes, immunohistochemistry (CD31, prostaglandin endoperoxide synthase 1/PTGS1) and assessment of tumour vascularisation by conventional histopathology.

PTSMT showed similar or fewer vessels than leiomyomas. Of the genes under investigation, 23 were down-deregulated (pro-angiogenic and some anti-angiogenic factors) and five were up-regulated (e.g. PTGS1 which is expressed at very low levels in leiomyomas but moderately higher levels in PTSMT).

In summary, no particular target molecule could be identified, because tumour angiogenesis in PTSMT is characterised by low levels of major pro-angiogenic factors and there is no prominent increase in tumour vascularisation. EBV can induce angiogenesis via its viral late membrane protein 1 (LMP1) but PTSMT frequently do not express LMP1, which could be an explanation why, despite EBV infection, PTSMT show no exaggerated tumour angiogenesis.

## Introduction

Epstein-Barr virus (EBV)-associated post-transplant smooth muscle tumours (PTSMT) are rare complications following solid graft and stem cell transplantation [1]. The molecular pathobiology of this rare neoplastic entity is not fully understood and only few experimental analyses have addressed this issue [1,2]. Tumour cells are thought to be derived from aberrant myogenous venous/perivascular wall cells [3]. They express smooth muscle proteins (actin and desmin), but not CD117, CD34 or other endothelial marker proteins. Histomorphology is characterised by mild atypia, low mitotic rate and absence of prominent tumour necrosis. All in all, PTSMT show more histological features of benign leiomyomas rather than leiomyosarcomas [1,2] and our group has previously analysed cell cycle factors, cytokines and gene promoter methylation in PTSMT and found an activated phosphoinositide 3-kinase (PI3K)/mammalian target of rapamycin (mTOR) cell cycle pathway as well as expression of vascular endothelial growth factor (VEGF) and Fms-related tyrosine kinase 1 (FLT1/VEGFR1) [1]. In general, in addition to endogenous molecular defects which affect mitosis and apoptosis of the tumour cells, angiogenesis is a major mechanism which contributes to tumour cell survival by supplying the metabolism of aberrant cell proliferation. Currently, for PTSMT, surgery and reduced immunosuppression are the therapy of choice [1]. At this point, there is no proof that patients benefit from conventional chemotherapy or radiation alone [1]. In other soft tissue neoplasms, numerous studies have addressed the angiogenic microenvironment as a potential target of therapy. In PTSMT, angiogenesis might be of special importance, as the original/progenitor tumour cell in these neoplasms is generally thought to be derived from an aberrant perivascular/venous wall cell. This topic is also important in PTSMT, as these can manifest in any anatomical localisation and cerebral tumours are in particular associated with a poor prognosis [1].

From other tumours, in particular renal cell cancer, we know that hypoxia-inducible factor 1, alpha subunit (HIF1A) signalling mediates expression of VEGF, platelet-derived growth factor (PDGF) and angiopoietin via the PI3K/mTOR pathway [4,5]. These cytokines activate pro-angiogenic receptors such as VEGFR and PDGF receptors (PDGFR). For a variety of neoplasms, e.g. soft tissue sarcomas such as leiomyosarcomas, it has been shown that a VEGFR/PDGFR-mediated increase of angiogenesis can be inhibited by anti-angiogenic agents [6-9]. The aim of this analysis was to evaluate the expression pattern of angiogenesis-related genes in PTSMT, in order to identify potential target molecules for anti-angiogenic therapy, in particular for those patients who suffer from irresectable or progressive tumours.

## Material and methods

### Tissue specimens

Five EBV^+^ PTSMT samples from four patients, including two tumours from one patient (#4), and seven EBV^-^ benign uterine leiomyomas from solid graft recipients were analysed. These cases had been characterised earlier (Additional file 1: Table S1) [1]. Formalin-fixed and paraffin-embedded (FFPE) samples were retrieved from the archives of the Institute of Pathology (Hannover Medical School/MHH, Hanover, Germany). The retrospective evaluation has been approved by the local ethics committee (MHH).

### Expression analysis of angiogenesis-associated factors

Tissue from FFPE blocks with >90% tumour cells were cut and processed for further PCR analysis. In blocks with <90% aberrant neoplastic cells, the PTSMT compartments of the specimens were laser microdissected using a SmartCutPlus-System (MMI, Glattbrugg, Switzerland), as previously described [1,10]. Cells were digested in proteinase K and RNA was extracted with phenol/chloroform [1,10]. Synthesis of cDNA from mRNA, subsequent pre-amplification of cDNA and real-time quantitative PCR of 45 angiogenesis-associated genes and three endogenous controls with a 7900HT Fast Real-Time PCR system were performed according to the manufacturers’ instructions (Applied Biosystems, Carlsbad, CA, USA). Endogenous controls were polymerase (RNA) II (DNA-directed) polypeptide A, 220 kDa (POLR2), glucuronidase beta (GUSB) and glyceraldehyde-3-phosphate dehydrogenase (GAPDH). Delta C_T_ values were converted into 2^-ΔCT^ values (normalised to a mean of endogenous control genes). Statistical analysis was performed with Prism 5.0 (GraphPad Software, San Diego, CA, USA) by applying the non-parametric Kruskal-Wallis test followed by the Mann-Whitney test for two-group comparison. P values < 0.05 were considered as statistically significant.

### Immunohistochemistry for evaluation of selected genes

Deparaffinised and rehydrated FFPE tissue sections (1-2 μm) were stained after autoclave pre-treatment. For staining of platelet/endothelial cell adhesion molecule 1 (VCAM1/CD31), sections were processed in an automated staining system (Benchmark ULTRA, Ventana Medical Systems, Inc., Tucson, AZ, USA). Prostaglandin endoperoxide synthase 1 (prostaglandin G/H synthase and cyclooxygenase) (PTGS1) was stained manually (positive control: FFPE prostate cancer tissue). Mouse monoclonal antibodies were used. Vascularisation was quantified by counting CD31^+^ vessels per 10 high power fields (HPF) and then correlating them in serially cut haematoxylin-eosin-stained sections. Statistical analysis was performed with Prism 5.0 as described above.

## Results

### Vascularisation of PTSMT

As previously described, PTSMT tumour cells themselves were negative for CD31. In the cerebral PTSMT we could previously demonstrate aneuploidy of the MYC locus 8q24 by fluorescence *in situ* hybridisation (FISH) [1]. In this case, endothelial cells showed a normal MYC configuration. Thus, a clonal relation between PTSMT and endothelial cells could not be proven (Additional file 1: Figure S1).

**Figure 1:**
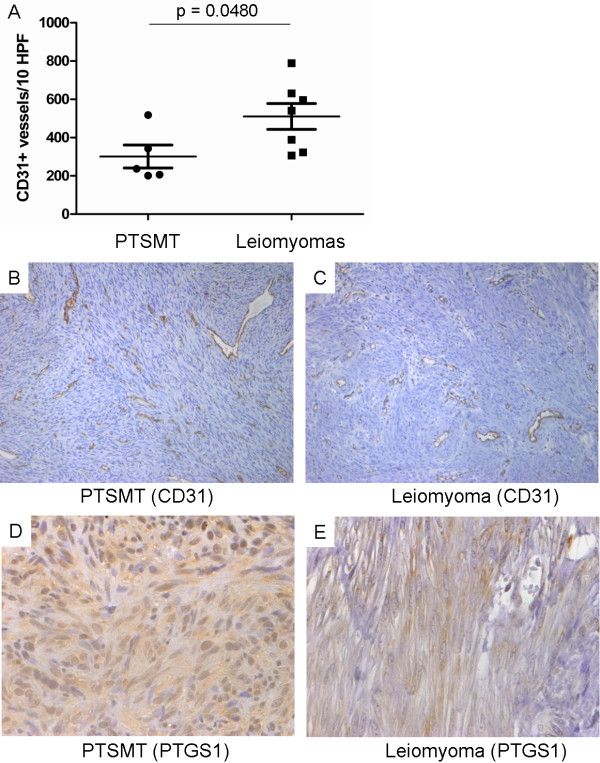
**Similar vascularisation of PTSMT and leiomyomas. A)** Histological counts of vessels. **B)** CD31+ vessels in PTSMT (original magnification x200). **C)** CD31+ vascularisation in leiomyomas (x200). **D)** Weak PTGS1 protein expression in PTSMT (x400). **E)** Very weak protein expression in a leiomyoma (x400).

PTSMT showed similar or fewer vessels than leiomyomas (mean 301/range 201-518 versus mean 511/range 306-789 CD31^+^ vessels/10 HPF, p = 0.0480; Figure 1). Corresponding to the low significance level, there was a broad overlap in vessel density between these two leiomyomatous tumour entities. Furthermore, gene expression analysis of CD31 did not correlate with vessel density. Higher rather than lower expression levels of CD31 were detectable in PTSMT (mean 20.10/range 5.26-30.48 in PTSMT versus mean 6.76/range 2.44-11.40 in leiomyomas; p = 0.0303). Sinusoids without smooth muscle cell wall appeared generally smaller in PTSMT and more hyalinised but, in comparison to leiomyomas the quantitative difference was not significant. PTSMT had significantly fewer arterioles, as defined by vessels with a smooth muscle wall (mean 1 versus 15 vessels/10 HPF, p = 0.0058). In summary, there was no clear evidence that PTSMT are generally more vascularised than leiomyomas.

### Reduced expression of angiogenesis-associated genes in PTSMT

**Figure 2:**
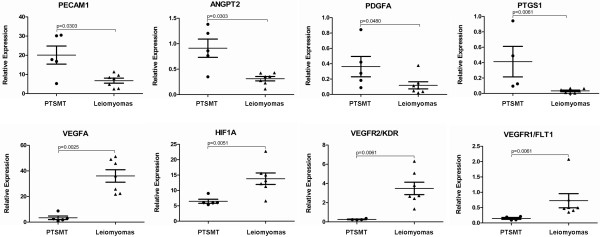
Representative summary of up- and down-regulated angiogenesis-associated factors in PTSMT.

Among 45 angiogenesis-associated mediators under investigation, 28 were significantly deregulated in PTSMT: 23 were down-deregulated and 5 (including CD31, see above) were up-regulated (Figure 2, Table 1).

**Table 1:** Expression profile of angiogenesis-related genes

**Gene (abbreviation)**	**Gene name**	**PTSMT (mean)**	**Leiomyomas (mean)**	**Significance**
ANGPT1	Angiopoietin 1	0.03	0.51	n.s.
ANGPT2	Angiopoietin 2	0.91	0.31	p = 0.0303
ANGPTL1	Angiopoietin-like 1	0.05	0.05	n.s.
ANGPTL4	Angiopoietin-like 4	0.03	0.05	n.s.
BAI1	Brain-specific angiogenesis inhibitor 1	0.06	0.18	n.s.
CAV2	Caveolin 2	0.84	2.35	n.s.
CDK1	Cyclin-dependent kinase 1	0.01	0.01	n.s.
CXCL1	Chemokine (C-X-C motif) ligand 1 (melanoma growth stimulating activity, alpha)	4.25	7.55	n.s.
EPHB4	EPH receptor B4	0.06	2.50	p = 0.0025
FGF2	Fibroblast growth factor 2 (basic)	0.01	0.16	p = 0.0121
FGFR1/FLT2	Fibroblast growth factor receptor 1	3.36	21.33	p = 0.0051
GREM1	Gremlin 1, DAN family BMP antagonist	0.14	0.20	n.s.
HGF	Hepatocyte growth factor	0.02	0.07	p = 0.0242
HIF1A	Hypoxia inducible factor 1, alpha subunit	6.48	13.81	p = 0.0051
IL8	Interleukin 8	0.60	0.03	n.s.
LECT1	Leukocyte cell derived chemotaxin 1	0.11	Not detectable	--
MDK	Midkine (neurite growth-promoting factor 2)	0.14	1.24	p = 0.0061
MMP2	Matrix metallopeptidase 2 (gelatinase A, 72 kDa gelatinase, 72 kDa type IV collagenase)	1.19	34.07	p = 0.0025
NOS3	Nitric oxide synthase 3 (endothelial cell)	0.05	0.31	p = 0.0025
NOTCH4	Notch 4 (receptor)	0.28	2.68	p = 0.0025
NOX4	NADPH oxidase 4	0.05	1.32	p = 0.0061
NRP1	Neuropilin 1	0.17	0.82	n.s.
OLR1	Oxidized low density lipoprotein (lectin-like) receptor 1	0.04	0.02	n.s.
PDGFA	Platelet-derived growth factor alpha polypeptide	0.36	0.12	p = 0.0480
PDGFRA	Platelet-derived growth factor receptor, alpha polypeptide	1.66	7.31	n.s.
PDPN	Podoplanin	0.09	3.36	p = 0.0061
PECAM1/CD31	Platelet/endothelial cell adhesion molecule	20.10	6.76	p = 0.0303
PTGS1	Prostaglandin-endoperoxide synthase 1 (prostaglandin G/H synthase and cyclooxygenase)	0.41	0.03	p = 0.0061
PTRF	Polymerase I and transcript release factor	7.68	56.80	p = 0.0025
S1PR1	Sphingosine-1-phosphate receptor 1	0.15	0.76	p = 0.0172
SERPINF1	Serpin peptidase inhibitor, clade F (alpha-2 antiplasmin, pigment epithelium derived factor), member 1	0.19	6.45	p = 0.0061
STAB1	Stabilin 1	0.06	0.16	n.s.
TEK	TEK tyrosine kinase, endothelial	0.11	1.17	p = 0.0043
TGFBR1	Transforming growth factor, beta receptor 1	0.05	0.38	p = 0.0025
THBS1	Thrombospondin 1	13.56	134.17	p = 0.0025
TIMP2	TIMP metallopeptidase inhibitor 2	9.32	54.78	p = 0.0025
TNFAIP2	Tumor necrosis factor, alpha-induced protein 2	0.08	0.01	n.s.
TYMP	Thymidine phosphorylase	0.17	0.08	p = 0.0480
VCAM1	Vascular cell adhesion molecule 1	0.18	0.95	p = 0.0303
VEGFA	Vascular endothelial growth factor A	3.38	36.05	p = 0.0025
VEGFC	Vascular endothelial growth factor C	0.47	1.66	p = 0.0043
VEGFD/FIGF	C-fos induced growth factor (vascular endothelial growth factor D)	0.01	0.23	n.s.
VEGFR1/FLT1	Fms-related tyrosine kinase 1 (vascular endothelial growth factor/vascular permeability factor receptor)	0.15	0.73	p = 0.0061
VEGFR2/KDR	Kinase insert domain receptor (a type III receptor tyrosine kinase)	0.26	3.48	p = 0.0061
VEGFR3/FLT4	Fms-related tyrosine kinase 4	0.08	0.10	n.s.

Prominently down-regulated factors included e.g. pro-angiogenic HIF1A, fibroblast growth factor receptor 1 (FGFR1/FLT2), kinase insert domain receptor (VEGFR2/KDR) and VEGFA as well as anti-angiogenic serpin peptidase inhibitor, clade E (nexin, plasminogen activator inhibitor type 1), member 1 (SERPINE1), thrombospondin 1 (THBS1) and TIMP metallopeptidase inhibitor 2 (TIMP2).

Except for CD31, significant differences of other up-regulated factors were due to very low expression in leiomyomas rather than strong expression in PTSMT. These factors were angiopoietin 2 (ANGPT2), PDGFRA, PTGS1 and thymidine phosphorylase (TYMP). Because PTGS1 can be inhibited by widely used non-steroidal anti-inflammatory drugs, immunohistochemistry was performed for evaluation if the tumour cells showed a corresponding protein expression. A weak expression of PTGS1 proteins in PTSMT and leiomyomatous smooth muscle spindle cells was detectable (Figure 1). Weak protein expression corresponded with relatively low transcript expression levels in both tumour types (mean 0.41/range 0.09-0.94 in PTSMT versus mean 0.03/range 0.00-0.08 in leiomyomas; p = 0.0061).

## Discussion

Patients suffering from PTSMT benefit from surgical tumour resection and/or reduction of immunosuppression [1]. However, surgical respectability depends on tumour site and, of note, PTSMT can manifest at any localisation, including the transplanted organ, in particular liver grafts [1]. Furthermore, multiple PTSMT, e.g. in the lung, are not suitable for a surgical approach [11]. Due to the rarity of this tumour entity, prospective evaluations of therapeutic strategies will not be applicable in a considerable number of patients. However, additional therapy options are mandatory for those patients who cannot be operated and/or whose transplant organ does not tolerate reduction of immunosuppression. In individual patients, it has been shown that inhibition of mTOR signal pathways by sirolimus might be of therapeutic benefit [12-14]. The rationale for administration of an mTOR signalling inhibitor was based on the finding that PTSMT and HIV-associated SMT, which share morphological similarities with PTSMT, express mTOR [2]. However, sirolimus cannot be administered to all transplanted patients, e.g. after renal transplantation, because the drug is potentially nephrotoxic. Another class of drugs which is widely used for systemic therapy of soft tissue neoplasms/sarcomas are anti-angiogenic agents, e.g. leiomyosarcoma [8]. Basic analysis of tumour-associated angiogenesis is important for assessing the vulnerability of a given tumour type to these drugs. Prominent proliferation of vessels, high expression levels of pro-angiogenic and low levels of anti-angiogenic genes would make it likely that PTSMT patients could benefit from anti-angiogenic drug therapy. Therefore, we evaluated the expression profiles of angiogenesis-related factors in PTSMT. However, in contrast to this assumption we found almost the opposite: PTSMT showed similar or even reduced vascularisation, when compared to sporadic leiomyomas. Furthermore, we could show that this morphological feature was based on a previously unknown molecular characteristic of PTSMT, namely expression of low levels of pro-angiogenic factors and high levels of anti-angiogenic genes. In particular major factors of hypoxia-inducible angiogenesis such as HIF1A, VEGFA, VEGFC, VEGFR1/FLT1, VEGFR2/KDR and FGFR1/FLT2 were expressed at low levels. In contrast to PTSMT, leiomyosarcomas show generally higher expression of VEGFA than leiomyomas [15-17]. In leiomyosarcoma-derived cell lines it could be demonstrated that hepatocyte growth factor (HGF) induces a decrease in anti-angiogeneic THBS1 and an increase in VEGFA [18]. In PTSMT, HGF, THBS1 and VEGFA are all expressed at low levels, indicating that HGF signalling does not contribute significantly to tumour angiogenesis. In PTSMT, low levels were also detectable for other pro-angiogenic genes which are involved in differentiation and proliferation of endothelial cells, e.g. vascular development-related EPH receptor B4 (EPHB4) and sphingosine-1-phosphate receptor 1 (S1PR1), the endothelium-specific receptor tyrosine kinase TEK and the growth factor midkine (MDK). Immune response-associated caveolae are plasma membrane invaginations of 60-80 nm in diameter in endothelial cells, smooth muscle cells and other cell types [19] and caveolae components CAV2 (p = 0.07) and PTRF (p < 0.01) were both decreased in PTSMT. In addition to several blood vessel-associated factors, lymphatic vessel protein podoplanin was decreased in PTSMT. Again, in leiomyosarcomas, podoplanin-positive vessels are especially found in tumours with lymph node metastases [20]. In our cohort, none of the PTSMT manifested in lymph nodes and, in general, involvement of lymph nodes is rare in this type of transplant-associated neoplasm [1]. MMP2, which degrades the collagen IV-rich basal membrane as a necessary requisite for metastasis [21], was reduced in PTSMT, which indicates no major remodelling of extracellular matrix during tumour cell and endothelial proliferation.

Compared to leiomyomas, only a few pro-angiogenic factors such as TYMP, ANGPTL2 and PTGS1 were increased in PTSMT. However, statistical significances were the result of very low expression levels in leiomyomas rather than a prominent up-regulation in PTSMT. The mean relative expression levels of these three factors was <1, indicating no major role in mediating tumour angiogenesis.

In PTSMT, three important anti-angiogenetic factors were decreased: TIMP2, SERPINF1 and THBS1. TIMP2 and SERPINF1 are strong inhibitors of endothelial proliferation [8,22] and THBS1 induces reduced migration ability of endothelial cells [23]. Furthermore, THBS1 can inhibit the binding of activating cytokines at receptors of endothelial cells and can also bind to the thrombospondin receptor CD36 which induces endothelial apoptosis [23]. Other groups found that leiomyomas express THBS1 more frequently than leiomyosarcomas [24]. In addition, TIMP2 is also expressed at relatively low levels in leiomyosarcomas [22].

It has been shown that the transcription factor MYC leads to expression of the chromosome segment 13q31.3-encoded microRNA 17~92 cluster which includes the two paralogues miR-19a and miR-19b-1 [25-27]. MicroRNA are non-coding molecules of 20-25 nucleotides which bind to mRNA and negatively regulate protein translation [28]. THBS1-mRNA has a miR-19 binding site and therefore MYC-related miR-19 expression down-regulates THBS1 [25-27]. PTSMT have an increased MYC expression [1] and low levels of THBS1 but no up-regulation of the miR 17~92 cluster, including miR-19a (in PTSMT mean relative expression level 0.02 versus 0.03 in leiomyomas) and miR-19b (mean 1.63 in PTSMT versus 2.23 in leiomyomas) [29]. The microRNA profile in PTSMT is overall associated with leiomyomatous differentiation of the tumour cells [29]. Therefore, similar to mesenchymal cells *in vitro* and *in vivo*[30], in PTSMT increased MYC expression is associated with decreased THBS1 expression but there is no indication for a specific microRNA regulation. Furthermore, while in leiomyosarcomas low expression of THBS1 and TIMP2 is accompanied by increased expression of pro-angiogenic factors such as VEGFA, PTSMT in general did not show such a global pro-angiogenic expression profile.

As reviewed by Paydas [31], in lymphomas and nasopharyngeal carcinomas, tumour cell infection with EBV is related to increased angiogenesis, in particular because the viral late membrane protein 1 (LMP1) induces expression of VEGF and activation of PTGS2, interleukin 8 (IL8), fibroblast growth factor 2 (FGF2) and other pro-angiogenic factors. Although PTSMT are infected with EBV, these tumours do not usually express LMP1 proteins [1,2,32] and this could be an explanation why, despite viral infection, PTSMT show no exaggerated tumour angiogenesis. It is not known how the EBV+ tumour cells suppress the expression of LMP1 while expressing other viral proteins and the tumour biological benefit of this selective lack of LMP1 for the PTSMT proliferation is also not known.

Although clinical testing has not yet been performed, on the one hand it is questionable whether patients who suffer from this type of soft tissue tumour might benefit from systematic anti-angiogenic drug therapy. On the other hand, it could be assumed that PTSMT found their own equilibrium of tumour vascularisation that allows survival and growth without increasing the expression of pro-angiogenic factors (e.g. due to aberrant signalling downstream of pro-angiogenic receptors). This might principally indicate a limited ability to circumvent therapy and therefore anti-angiogenic drugs might not necessarily be ineffective since this would disrupt the equilibrium of PTSMT vascularisation. Anti-angiogenic drugs could still be administered to PTSMT patients with no other treatment options available but, in these present analyses, we could not identify a specific target molecule.

In summary, our analyses of the tumour angiogenesis in PTSMT revealed no particular target molecule, because PTSMT are characterised by low levels of major pro-angiogenic factors and there is no prominent increase in tumour vascularisation.

## Competing interests

The authors of this manuscript have no conflicts of interests to disclose.

## Authors’ contributions

Histomorphology (DJ, KH, HK), molecular analysis (LM, NI, ES), data collection, analysis of data and manuscript preparation (DJ, LM, NI, ES, HK, KH). All authors read and approved the final manuscript.

## Supplementary Material

Additional file 1: Table S1 Patient cohort. **Figure S1.** Fluorescence in situ hybridisation of the MYC gene shows no aneuploidy in endothelial cells (white arrows) while a subfraction of PTSMT cells had an aneuploidy. Click here for file
