# Supplementary material for: Tumour angiogenesis in Epstein-Barr virus-associated post-transplant smooth muscle tumours
Source: Clin Sarcoma Res. 2014 Jan 7;4:1. doi: 10.1186/2045-3329-4-1 (PMC3896710; doi:10.1186/2045-3329-4-1)
Supplement: Additional file 1: Table S1 — Patient cohort. Figure S1. Fluorescence in situ hybridisation of the MYC gene shows no aneuploidy in endothelial cells (white arrows) while a subfraction of PTSMT cells had an aneuploidy. [file 2045-3329-4-1-S1.doc]

Supplementary Table 1

|  | **Gender, age** | **Transplant organ** | **Tumour manifestation and localisation** |
| --- | --- | --- | --- |
| **#1** | ♀  6 years | Liver | +16 months after transplantation, EBV^+^ PTSMT (liver) |
| #2 | ♀  7 years | Liver | +68 months after transplantation, EBV^+^ PTSMT (bronchus) |
| **#3** | ♀  15 years | Heart | +28 months after transplantation, EBV^+^ PTSMT (colon) |
| **#4** | ♀  13 years  (Fanconi anaemia) | Bone marrow | +52 months after transplantation, two EBV^+^ PTSMT (cerebral sinus/#4-1 and spleen/#4-2) |
| **#5-#11** | 7 ♀  Median 39  (32-55) | Kidney | +82 months after transplantation, EBV^-^ leiomyomas (uterus), 55-year-old patient |
|  |  | No transplantation | EBV^-^ leiomyomas (uterus), six patients |

**Supplementary Figure 1**

**
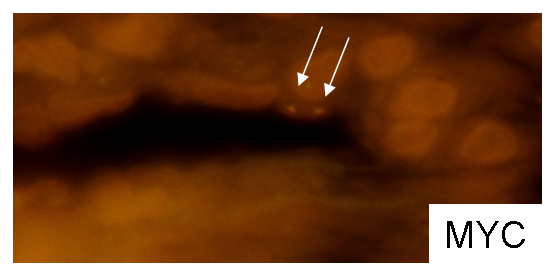
**

**Legend to supplementary Figures and Tables**

**Supplementary Figure 1**

**Fluorescence in situ hybridisation of the MYC gene shows no aneuploidy in endothelial cells (white arrows) while a subfraction of PTSMT cells had an aneuploidy.**

**Supplementary Table 1**

**Patient cohort.**
